# Development and implementation of a machine learning-based tool for predicting in-hospital adverse events in coronary heart disease

**DOI:** 10.1186/s12872-026-06376-6

**Published:** 2026-09-09

**Authors:** Xiyun Zhang, Yi Zhang, Peng Zhao

**Affiliations:** 1 School of Science, Shijiazhuang University, Shijiazhuang, Hebei China; 2 The Smart Hospital Construction Department, The First Hospital of Hebei Medical University, Shijiazhuang, Hebei China

**Keywords:** Coronary Heart Disease, In-Hospital Adverse Events, Machine Learning, Random Forest, Clinical Decision Support System, Web Application, Causal Inference, Risk Prediction

## Abstract

**Background:**

Coronary Heart Disease (CHD) is a leading cause of mortality worldwide. In-hospital adverse events remain a critical challenge affecting patient outcomes. Early identification of high-risk patients using predictive models could improve clinical outcomes.

**Objective:**

To develop and validate a machine learning-based prediction model for in-hospital adverse events in CHD patients, explore causal effects of key risk factors, and implement the model as a web-based clinical decision support tool.

**Methods:**

This retrospective cohort study included 1,288 CHD patients who underwent cardiac surgery at a tertiary hospital from January 2018 to December 2023. Patient demographics, laboratory test data (233 items with coverage rate $\ge 5\%$), surgical information, and in-hospital adverse events were collected. Variables with more than 60% missingness were removed, and patients with more than 10% missingness across the retained predictors were excluded from the primary analysis. After the training–test split, remaining item-level missingness was handled using MICE in the training set and training-derived median or mode imputation in the test set. LASSO regression was used for feature selection. Six machine learning algorithms (XGBoost, Random Forest, Logistic Regression, SVM, LightGBM, and Ensemble Model) were compared for prediction performance. Causal forest method was employed to explore the causal effects of key variables on adverse events. Model performance was evaluated using Area Under the Receiver Operating Characteristic Curve (AUC), accuracy, sensitivity, specificity, and F1-score. The optimal model was implemented as a web application using R Shiny framework.

**Results:**

Among 1,288 patients, 272 (21.1%) experienced in-hospital adverse events. After LASSO feature selection within the training set, 9 key predictor variables were retained, including neutrophil percentage, albumin, bleeding volume, albumin/globulin ratio, and neutrophil absolute count. The Random Forest model achieved the best overall performance with an AUC of 0.7418 (95% CI: 0.6847–0.7990), sensitivity of 67.07%, specificity of 66.12%, and accuracy of 66.32%. Causal forest analysis demonstrated that red blood cell count had a protective effect, reducing adverse event risk by 6.76% (95% CI: 1.38–12.14%, *p*=0.014). The developed web application provides real-time risk assessment with model interpretability features, enabling clinicians to input patient data and obtain immediate, calibrated risk estimates with clinical recommendations.

**Conclusion:**

The Random Forest model demonstrated moderate discrimination for predicting in-hospital adverse events in CHD patients undergoing cardiac surgery. Causal forest analysis suggested that higher red blood cell count was associated with a lower estimated risk, whereas the effects of the other evaluated predictors were not statistically significant. The web application illustrates the potential clinical translation of the model but requires external and prospective validation before routine use.

## Introduction

Coronary Heart Disease remains the leading cause of mortality and morbidity worldwide, accounting for approximately 17 million deaths annually according to the American Heart Association statistics [1]. The prevalence of CHD continues to rise, particularly in aging populations, creating substantial burdens on healthcare systems. Despite significant advances in percutaneous coronary intervention and coronary artery bypass grafting, in-hospital adverse events remain a critical challenge affecting patient outcomes, prolonging hospital stays, and increasing medical costs.

In-hospital adverse events in CHD patients encompass a spectrum of complications including cardiovascular events such as arrhythmia, heart failure, and cardiogenic shock, bleeding complications, infections, acute kidney injury, and respiratory failure. The incidence of these adverse events ranges from 15 to 30 percent depending on patient populations and definitions used in different studies [2, 3]. These complications not only increase patient suffering but can also lead to increased mortality, with some studies reporting in-hospital mortality rates up to 5 percent in high-risk patients [4, 5]. Therefore, early identification of high-risk patients and implementation of targeted preventive interventions have important clinical significance.

Previous epidemiological studies have identified multiple risk factors for adverse events in cardiac surgery patients, including advanced age, left ventricular dysfunction, chronic kidney disease, diabetes, and emergency surgery status [6, 7]. However, traditional risk assessment tools such as the European System for Cardiac Operative Risk Evaluation and the Society of Thoracic Surgeons risk score, while useful, may not fully capture the complex interactions among contemporary clinical variables and laboratory parameters [8]. These scoring systems are predominantly based on traditional statistical methods such as logistic regression and incorporate only a limited number of clinical variables, making it challenging to fully reflect the complex pathological status of patients in the era of big data [9, 10].

Several limitations constrain the utility of traditional scoring systems. First, they assume linear relationships between predictors and outcomes, which may fail to capture complex nonlinear associations and interaction effects that exist in physiological systems [11, 12]. Second, traditional methods struggle to effectively process high-dimensional laboratory test data containing hundreds of variables [13, 14]. Machine learning techniques have shown considerable potential in medical research over the past decade [1, 15]. Compared with conventional statistical models, machine learning methods can flexibly capture nonlinear relationships and complex interactions among high-dimensional predictors, while regularization and cross-validation can reduce overfitting. The missing-data preprocessing procedures used in this study are also applicable to conventional regression models; therefore, the principal motivation for using machine learning was its flexibility in modeling nonlinear and interactive effects rather than any inherent advantage in handling missing data. Recent studies have demonstrated that machine learning approaches can outperform traditional scoring systems in predicting cardiac surgery outcomes [16, 17]. A systematic comparison by Allyn and colleagues found that machine learning models achieved better discrimination compared with EuroSCORE II in predicting postoperative mortality. Similarly, Churpek and colleagues showed that machine learning methods outperformed traditional logistic regression in predicting cardiac arrest in hospitalized patients [4].

Machine learning techniques have shown powerful application potential in the medical field over the past decade [1, 15]. Compared with traditional statistical methods, machine learning algorithms offer several advantages including the capability to automatically process high-dimensional data and mine potential nonlinear relationships and interaction effects, effective avoidance of overfitting through cross-validation and regularization, provision of feature importance rankings to help identify key predictors, and provision of feature importance rankings to help identify key predictors [18, 19]. The missing data preprocessing steps employed here are equally applicable to traditional regression models; the primary advantage of ML lies in capturing non-linear relationships and complex interactions among high-dimensional variables. Various machine learning algorithms have been applied to cardiovascular disease prediction, including XGBoost and LightGBM as gradient boosting decision tree methods that have demonstrated excellent performance in multiple prediction tasks, Random Forest as an ensemble learning method that provides robust predictions and interpretable feature importance, and Support Vector Machines that can handle complex boundary conditions in classification tasks [20–22]. Comparative studies have shown that gradient boosting methods generally outperform other algorithms in cardiovascular risk prediction [23, 24].

Beyond prediction accuracy, interpretability remains a challenge for clinical implementation. Recent advances in explainable AI, including SHAP and LIME, have facilitated the translation of machine learning models into clinical practice [11]. Furthermore, causal inference methods have gained increasing attention in cardiovascular research. Traditional observational studies can identify associations but cannot establish causal relationships due to confounding. Causal inference methods, including causal forests and doubly robust estimators, can estimate treatment effects from observational data while addressing confounding bias. The causal forest method, developed by Wager and Athey and extended by Athey and Imbens, provides a powerful tool for estimating heterogeneous treatment effects from observational data. This approach can identify subgroups of patients who may benefit more or less from specific interventions, supporting personalized clinical decision-making [25, 26]. Proper handling of missing data is critical for valid causal inference. Multiple Imputation by Chained Equations has become a standard approach for handling missing data in clinical research, producing valid estimates under the missing at random assumption. Studies have demonstrated that MICE combined with machine learning can improve both prediction accuracy and causal effect estimation [27].

Despite the growing literature on machine learning for cardiovascular risk prediction, several gaps remain in the current literature [4]. Limited studies have combined machine learning with causal inference to explore risk factors. Most studies focus on mortality rather than broader adverse events [5, 21]. Few studies have incorporated the full spectrum of laboratory test data. Validation studies in Chinese populations are scarce [3, 16]. This study aims to address these gaps by constructing a machine learning prediction model for in-hospital adverse events using comprehensive clinical data from CHD patients, comparing the predictive performance of multiple machine learning algorithms and selecting the optimal model, exploring causal effects of key risk factors using causal forest method, and providing a theoretical basis for clinical early warning and personalized intervention strategies [22, 24, 27].

## Methods

### Study design and population

This was a single-center retrospective cohort study conducted at a tertiary hospital in China. The study protocol was approved by the hospital Ethics Committee (Clinical Trial Number: 2025 Research Ethics Approval No. (S01104), Version: V2.0 (February 1, 2024)), with informed consent waived due to the retrospective nature of the study. The study followed the Strengthening the Reporting of Observational Studies in Epidemiology (STROBE) guidelines.

Data were collected from the inpatient information system of the Department of Cardiac Surgery from January 2018 to December 2023. The dataset included ten modules: patient basic information (demographic characteristics including age, gender, height, weight), diagnosis information (ICD-10 codes for CHD: I20-I25), smoking and drinking history, past medical history, comorbidity information (hypertension, diabetes), admission information, vital signs (respiration rate, pulse, blood pressure), laboratory test data (233 test items), in-hospital adverse events as the outcome variable, and surgical information (operation duration, bleeding volume, anesthesia method).

Patients were eligible for inclusion if they were aged 18 years or older, had at least one confirmed diagnosis of CHD recorded using ICD-10 codes I20–I25, underwent cardiac surgery, and had non-missing age, sex, operation information, and in-hospital adverse-event status. The following sequential exclusions were applied:

- **Step 1 – Variable-level quality filtering**: Variables with >60% missing values were removed (no patient exclusion).
- **Step 2 – CHD diagnosis confirmation**: All 2,885 patients met the prespecified CHD diagnostic criterion; therefore, no additional patients were excluded at this step.
- **Step 3 – Patient-level missing-data filtering**: Patients with more than 10% missingness across the retained predictors were excluded from the primary analysis (*n = 1,204*). This prespecified quality-control threshold was used to limit reliance on imputation for patient records with extensive missing information, because imputing a substantial proportion of an individual record could make the completed data strongly dependent on the imputation model. The influence of this threshold was examined in expanded-cohort sensitivity analyses.
- **Step 4 – Sequential exclusion based on physiologically implausible values**: Physiologically implausible measurements were identified using prespecified thresholds. Among patients remaining after Step 3, the criteria were applied sequentially; once a patient was excluded at an earlier step, that patient was not counted again at subsequent steps. The thresholds were defined a priori as follows: age *<18* or *≥ 120* years; respiratory rate *<4* or *>60* breaths/min; heart rate or pulse *<20* or *>300* beats/min; systolic blood pressure *<40* or *>300* mmHg; and diastolic blood pressure *<20* or *>200* mmHg.Additional prespecified thresholds were height *<100* or *>220* cm, weight *<30* or *>200* kg, body mass index *<15* or *>50* kg/m$^2$, and operation duration *<30* or *>600* minutes.

The outcome variable was defined as in-hospital adverse event, which encompassed any adverse clinical event occurring during hospitalization, including cardiovascular complications (arrhythmia, heart failure, cardiogenic shock, myocardial infarction), bleeding events requiring intervention, infections (pneumonia, surgical site infection, sepsis), acute kidney injury, and respiratory failure. This was encoded as a binary variable (1 = adverse event, 0 = no adverse event).

A comprehensive set of predictor variables was collected, including demographic characteristics (gender, age), lifestyle factors (smoking status, drinking status), vital signs (respiration rate, pulse, systolic and diastolic blood pressure, height, weight, BMI), past medical history (cardiovascular surgery history, valvular heart disease history), comorbidities (hypertension, diabetes), laboratory test indicators (233 test items with coverage rate greater than or equal to 5 percent), and surgery-related variables (operation duration, bleeding volume, anesthesia method, surgery level).

Data processing was performed using R language (version 4.3.0). Laboratory test data were left-joined with basic information using patient ID to construct a wide-format dataset. For patients with multiple test results, the latest record was retained to ensure temporal consistency. Variables with more than 60% missing observations were removed. For the primary analysis, patients with more than 10% missingness across the retained predictors were excluded.

Before any data-driven imputation or feature selection, the resulting primary-analysis cohort was randomly divided into training and held-out test sets at a 7:3 ratio using stratified sampling according to the outcome. The training set (*n = 903*) was used for imputation-model estimation, feature selection, model development, and hyperparameter tuning. The test set (*n = 385*) was reserved exclusively for final performance evaluation.

Within the training set, remaining item-level missing values were imputed using Multiple Imputation by Chained Equations. Five imputed datasets were generated with five iterations. Variable-specific methods were assigned according to variable type and number of categories: predictive mean matching was used for numerical variables, logistic regression for binary factors, and polytomous logistic regression for factors with three to ten observed levels; factors with more than ten observed levels were handled using predictive mean matching. The outcome variable was not imputed. Median or mode imputation was used only as a fallback for any residual missing values remaining after MICE.

The held-out test set was not used to estimate the MICE models. Missing numerical and categorical values in the test set were replaced using medians and modes, respectively, calculated from the original training data. Thus, no test-set information was used to determine the imputation values.

Missing value imputation was performed using Multiple Imputation by Chained Equations (MICE). Variables with high missing rate (greater than 10%) were directly deleted, while low missing rate variables were imputed using MICE with 10 iterations. Categorical variables were imputed with mode, and numerical variables with median. Importantly, MICE was applied only to the training set. The test set was imputed separately using the median/mode values derived from the training set, ensuring that no information from the test set influenced the imputation process. All categorical variables were converted to factor type, and numerical variables remained continuous. All numerical variables were standardized using z-score normalization based on training set mean and standard deviation.

The dataset was first randomly divided into training and test sets at a 7 : 3 ratio using stratified sampling, ensuring consistent adverse event proportions in both sets. The training set (*n = 903*) was used for model training, feature selection, and hyperparameter tuning, while the test set (*n = 385*) was held out completely and used only for final performance evaluation.

Feature selection was performed using LASSO (Least Absolute Shrinkage and Selection Operator) regression as described by Tibshirani, applied exclusively within the training set. LASSO applies L1 regularization that encourages sparsity by shrinking less important coefficients to exactly zero. The optimal regularization parameter lambda was selected through 10-fold cross-validation on the training set using the minimum criteria (lambda.min). Variables with non-zero coefficients at the optimal lambda were retained as final predictors for subsequent modeling.

Six machine learning algorithms were compared in this study. XGBoost is an extreme gradient boosting algorithm with regularization (learning rate 0.1, max depth 6) to prevent overfitting as described by Chen and Guestrin. Random Forest is an ensemble learning method based on decision trees (*ntree = 500*,$mtry = \sqrt{p}$) as described by Breiman. Logistic Regression served as the traditional statistical learning method baseline. Support Vector Machine (SVM) used the radial basis function kernel. LightGBM is a lightweight and efficient algorithm based on gradient boosting ($number \ leaves = 31, learning \ rate = 0.05$) as described by Ke et al. The Ensemble Model used simple averaging of predictions from all above models.

Grid search combined with 10-fold cross-validation was used to optimize hyperparameters. For each algorithm, the optimal parameter combination was selected based on cross-validated AUC. Model performance was evaluated using multiple metrics including AUC, Accuracy, Sensitivity (Recall), Specificity, and F1-Score. AUC was used as the primary evaluation metric as it is not affected by classification thresholds and can comprehensively assess model discrimination ability. Feature importance was analyzed using the Random Forest model, with the mean decrease in Gini index calculated to rank the importance of predictor variables.

Causal Forest method was used to estimate the Average Treatment Effect (ATE) of key predictor variables as described by Wager and Athey and Athey and Imbens. The causal forest algorithm uses a variant of random forests adapted for causal inference, which can estimate heterogeneous treatment effects across individuals (CATE). For variables with an established clinical threshold, the corresponding clinical cutoff was used; for example, neutrophil absolute count was dichotomized at $7.0\times 10^9$/L. For variables without a prespecified clinical threshold, the training-set median was used. The exact cutoff used for each variable is reported in the causal forest results table. The ATE was estimated as the difference in outcome probability between the two groups. Bootstrapped confidence intervals (1000 iterations) were computed, and two-sided tests with p less than 0.05 were considered statistically significant.

All analyses were performed using R version 4.3.0 [28]. The following contributed packages were used: tidyverse [29] and dplyr for data processing, glmnet [30] for feature selection, randomForest [31], xgboost [32], lightgbm [33], and e1071 [34] for machine learning, pROC [35] for model evaluation, grf [36] for causal inference, mice [37] for missing value handling, and ggplot2 [38] for visualization. Significance level was set at *alpha = 0.05*.

## Results

### Baseline characteristics

The study population was derived from patients who underwent surgery and had complete surgical duration records. The following sequential exclusion steps were applied:

1. **Initial surgical cohort**: All patients with a documented operative time (*N = 2,885*).
2. **Variable-level filtering**: Variables with >60% missing values were removed (no sample exclusion).
3. **Exclusion of high missing rate samples**: Patients with >10% missing values across all remaining variables were excluded (*n = 1,204*).
4. **Exclusion of critical variable missing**: Patients missing any of the key variables (*in_hospital_adverse_event*, *Age*, *Gender*) were excluded (*n = 0*).
5. **Sequential exclusion of physiologically implausible values**: Among the 1,681 patients remaining after the patient-level missingness filter, the outlier criteria were applied sequentially. Once a patient was excluded at an earlier step, that patient was not assessed or counted again at subsequent steps. Therefore, the following counts represent additional, mutually exclusive exclusions at each step:
  Respiratory rate (*<4* or *>60* breaths/min): *n = 3*
  Diastolic blood pressure (*<20* or *>200* mmHg): *n = 1*
  BMI (*<15* or *>50* kg/m$^2$): *n = 4*
  Operation duration (*<30* or *>600* min): *n = 385*
  No additional patients were excluded at the age, heart rate or pulse, systolic blood pressure, height, or weight steps.

The initial cohort consisted of 2,885 patients. After variable-level filtering, 1,204 patients with more than 10% missingness across the retained predictors were excluded, leaving 1,681 patients. Across the initial cohort, 705 patients met at least one physiologically implausible-value criterion. Of these, 312 had already been included among the 1,204 patients excluded because of high patient-level missingness. Therefore, the sequential outlier-screening procedure resulted in 393 additional exclusions. The final primary-analysis cohort consequently consisted of 1,288 patients (*2,885-1,204-393=1,288*), including 1,016 patients without and 272 patients with an in-hospital adverse event.

Table 1 shows the baseline characteristics of the study subjects. Continuous variables are presented as median (Q1, Q3) and categorical variables as n (%). Significant differences (*p < 0.05*) were found between the adverse event and non-adverse event groups in the following variables:

**Table 1:** Baseline Characteristics by In-Hospital Adverse Event

Characteristic	Overall N = 1,288^1^	No N = 1,016^1^	Yes N = 272^1^	*p*-value^2^
**Demographics**				
Age (years)	66.0 (57.0, 71.0)	65.0 (57.0, 71.0)	67.0 (58.5, 74.0)	0.006
Gender				0.065
Male	865 (67%)	695 (68%)	170 (63%)	
Female	423 (33%)	321 (32%)	102 (38%)	
**Lifestyle**				
Smoking				0.5
No	1,144 (89%)	899 (88%)	245 (90%)	
Yes	144 (11%)	117 (12%)	27 (9.9%)	
Drinking				0.9
No	1,168 (91%)	922 (91%)	246 (90%)	
Yes	120 (9.3%)	94 (9.3%)	26 (9.6%)	
**Past Medical History**				
Past Medical History				0.002
No	610 (47%)	459 (45%)	151 (56%)	
Yes	678 (53%)	557 (55%)	121 (44%)	
**Comorbidities**				
Hypertension				0.6
FALSE	401 (31%)	320 (31%)	81 (30%)	
TRUE	887 (69%)	696 (69%)	191 (70%)	
Diabetes				0.3
FALSE	822 (64%)	641 (63%)	181 (67%)	
TRUE	466 (36%)	375 (37%)	91 (33%)	
**Vital Signs**				
Respiration (/min)	18.0 (18.0, 18.0)	18.0 (18.0, 18.0)	18.0 (18.0, 18.0)	0.035
Pulse (/min)	74.0 (66.0, 80.0)	74.0 (66.0, 80.0)	75.0 (68.0, 80.0)	0.3
Systolic_BP (mmHg)	133.0 (121.0, 145.5)	133.0 (122.0, 146.0)	131.0 (120.0, 143.0)	0.2
Diastolic_BP (mmHg)	80.0 (73.0, 88.0)	80.0 (74.0, 89.0)	80.0 (72.0, 87.0)	0.2
Height (cm)	168.0 (160.0, 172.0)	168.0 (160.0, 172.0)	165.5 (160.0, 170.0)	0.009
Weight (kg)	70.0 (62.5, 79.0)	70.0 (63.0, 80.0)	70.0 (60.0, 75.0)	0.010
BMI (kg/m^2^)	25.3 (23.1, 27.7)	25.3 (23.2, 27.8)	24.8 (22.8, 27.3)	0.2
**Surgery Information**				
operation_duration_min (min)	65.0 (47.0, 93.0)	64.0 (46.0, 90.0)	75.0 (50.0, 110.0)	<0.001
bleeding_volume (mL)	10.0 (5.0, 10.0)	10.0 (5.0, 10.0)	10.0 (5.0, 20.0)	0.004
Surgery Category				0.3
Formal_Surgery	976 (76%)	764 (75%)	212 (78%)	
Simple_Surgery	312 (24%)	252 (25%)	60 (22%)	
Anesthesia Type				<0.001
Non-General	1,060 (82%)	858 (84%)	202 (74%)	
General	228 (18%)	158 (16%)	70 (26%)	
**Blood Routine**				
White_Blood_Cell_Count (*× *10^9^/L)	6.0 (5.0, 7.4)	5.9 (4.9, 7.2)	6.2 (5.2, 8.1)	0.002
Neutrophil_Percentage (%)	65.0 (58.7, 71.4)	64.7 (58.2, 70.7)	66.9 (60.7, 75.5)	<0.001
Lymphocyte_Percentage (%)	24.5 (18.9, 30.6)	25.0 (19.4, 31.0)	22.9 (16.6, 29.1)	<0.001
Monocyte_Percentage (%)	7.2 (5.8, 8.4)	7.2 (5.9, 8.6)	7.0 (5.7, 8.2)	0.12
Eosinophil_Percentage (%)	1.8 (1.1, 3.0)	1.8 (1.1, 3.1)	1.7 (0.8, 2.6)	0.013
Basophil_Percentage (%)	0.4 (0.3, 0.7)	0.4 (0.3, 0.7)	0.4 (0.2, 0.6)	0.031
Neutrophil_Absolute_Count (*× *10^9^/L)	3.8 (3.1, 5.0)	3.8 (3.0, 4.8)	4.1 (3.3, 5.4)	<0.001
Lymphocyte_Absolute_Count (*× *10^9^/L)	1.4 (1.1, 1.8)	1.4 (1.1, 1.8)	1.3 (1.0, 1.8)	0.054
Monocyte_Absolute_Count (*× *10^9^/L)	0.4 (0.3, 0.5)	0.4 (0.3, 0.5)	0.4 (0.3, 0.6)	0.2
Eosinophil_Absolute_Count (*× *10^9^/L)	0.1 (0.1, 0.2)	0.1 (0.1, 0.2)	0.1 (0.1, 0.2)	0.034
Basophil_Absolute_Count (*× *10^9^/L)	0.0 (0.0, 0.0)	0.0 (0.0, 0.0)	0.0 (0.0, 0.1)	0.6
Red_Blood_Cell_Count (*× *10^12^/L)	4.4 (4.0, 4.7)	4.4 (4.0, 4.8)	4.3 (3.8, 4.6)	<0.001
Hemoglobin (g/L)	134.0 (122.0, 144.0)	135.0 (123.0, 144.0)	129.0 (118.0, 142.0)	<0.001
Hematocrit (%)	39.9 (36.3, 42.9)	40.2 (36.8, 43.0)	38.5 (34.8, 42.6)	<0.001
Mean_Corpuscular_Volume (fL)	90.9 (88.2, 93.9)	90.9 (88.2, 94.0)	91.0 (88.4, 93.7)	0.9
Mean_Corpuscular_Hemoglobin (pg)	30.5 (29.5, 31.6)	30.6 (29.5, 31.6)	30.5 (29.4, 31.7)	0.8
Mean_Corpuscular_Hemoglobin_Concentration (g/L)	336.0 (329.0, 341.0)	335.0 (329.0, 341.0)	336.0 (329.0, 341.0)	0.5
Red_Cell_Distribution_Width_CV (%)	13.4 (12.9, 13.9)	13.4 (12.9, 13.9)	13.4 (12.9, 14.0)	0.4
Red_Cell_Distribution_Width_SD (fL)	43.3 (41.6, 45.8)	43.3 (41.6, 45.5)	43.5 (41.6, 46.4)	0.6
Platelet_Count (*× *10^9^/L)	204.0 (173.0, 243.0)	205.0 (174.0, 243.0)	199.0 (169.5, 239.0)	0.2
Mean_Platelet_Volume (fL)	8.8 (8.0, 9.7)	8.8 (8.0, 9.7)	8.8 (8.0, 9.9)	0.2
Platelet_Distribution_Width (%)	16.7 (16.3, 17.1)	16.7 (16.3, 17.0)	16.7 (16.3, 17.1)	0.6
Plateletcrit (%)	0.2 (0.2, 0.2)	0.2 (0.2, 0.2)	0.2 (0.2, 0.2)	0.3
Platelet_to_Lymphocyte_Ratio (ratio)	143.3 (110.0, 188.3)	142.3 (109.3, 188.3)	146.9 (113.9, 189.7)	0.2
Neutrophil_to_Lymphocyte_Ratio (ratio)	2.7 (1.9, 3.8)	2.6 (1.9, 3.6)	3.0 (2.1, 4.6)	<0.001
Nucleated_RBC_Percentage (/100WBC)	0.1 (0.1, 0.1)	0.1 (0.1, 0.1)	0.1 (0.1, 0.1)	>0.9
**Coagulation**				
Thrombin Time (sec)	14.3 (13.6, 15.0)	14.3 (13.6, 14.9)	14.3 (13.7, 15.0)	0.5
**Biochemistry**				
Potassium (mmol/L)	4.0 (3.8, 4.3)	4.0 (3.8, 4.3)	4.0 (3.7, 4.2)	0.3
Sodium (mmol/L)	139.8 (138.4, 141.2)	139.8 (138.4, 141.3)	139.7 (138.4, 140.9)	0.2
Glucose (mmol/L)	5.6 (5.0, 7.0)	5.6 (4.9, 7.1)	5.6 (5.0, 6.9)	0.9
**Cardiac Biomarkers**				
B-type Natriuretic Peptide (pg/mL)	68.0 (28.8, 176.0)	66.2 (26.9, 174.0)	77.5 (36.1, 229.0)	0.009
**Lipid Profile**				
Lipoprotein (a) (mg/L)	146.4 (72.8, 366.0)	140.1 (73.8, 364.1)	170.6 (67.4, 398.6)	0.3

Patients with adverse events were older than those without adverse events (median 67.0 vs. 65.0 years, *p=0.006*). Although statistically significant differences were observed in height and weight, the absolute differences were small, and BMI did not differ significantly between the two groups. These anthropometric findings therefore appear to have limited standalone clinical relevance. The distribution of past medical history also differed between the groups (*p=0.002*), although this unadjusted comparison should not be interpreted as evidence of an independent association.

Regarding surgery-related indicators, patients in the adverse event group had longer operation duration (75.0 vs. 64.0 min, *p < 0.001*) and a higher proportion of general anesthesia (26% vs. 16%, *p < 0.001*).

Regarding laboratory indicators, the adverse event group showed significantly elevated white blood cell count (6.2 vs. 6.0 $\times 10^9$/L, *p = 0.002*), elevated neutrophil percentage (66.9% vs. 64.7%, *p < 0.001*), decreased lymphocyte percentage (22.9% vs. 25.0%, *p < 0.001*), elevated neutrophil-to-lymphocyte ratio (3.0 vs. 2.6, *p < 0.001*), elevated neutrophil absolute count (4.1 vs. 3.8 $\times 10^9$/L, *p < 0.001*), decreased hemoglobin (129.0 vs. 135.0 g/L, *p < 0.001*), decreased hematocrit (38.5% vs. 40.2%, *p < 0.001*), decreased red blood cell count (4.3 vs. 4.4 $\times 10^{12}$/L, *p < 0.001*), and higher B-type natriuretic peptide (77.5 vs. 66.2 pg/mL, *p = 0.009*).

For several variables, both group medians remained within the institutional reference range despite statistically significant differences, suggesting limited clinical importance when considered in isolation.

### Feature selection

To identify predictors of in-hospital adverse events, LASSO logistic regression with 10-fold cross-validation was applied within the training set (*N = 903*). The optimal tuning parameter $\lambda _{\min }$ was chosen via the minimum criteria, resulting in 9 non-zero coefficients. The selected variables and their coefficients are summarized in Table 2.

**Table 2:** Predictors selected by LASSO regression

Clinical predictor	LASSO coefficient
Monocyte percentage	*-0.005889*
Neutrophil absolute count	0.032454
Red blood cell count	*-0.028652*
Uric acid	*-0.000203*
Albumin (BCG method)	*-0.030662*
Albumin/globulin ratio	*-0.101809*
Operation duration	0.001898
Bleeding volume	0.000793
Male sex	*-0.042811*

Positive and negative coefficients indicate the direction of association in the penalized logistic regression model. Coefficient magnitudes should not be directly compared across predictors measured on different scales

Among the retained predictors, several reflected inflammatory status, including monocyte percentage and neutrophil absolute count. Albumin and the albumin/globulin ratio represented nutritional status, whereas red blood cell count reflected hematologic status. Uric acid represented a metabolic factor, and operation duration and bleeding volume reflected surgical complexity. Male sex was also selected. All nine predictors were retained for subsequent multivariable modeling.

### Model performance comparison

The prediction performance of 7 machine learning models on the test set is shown in Table 3.

**Table 3:** Performance Comparison of Different Models (95% CI in parentheses)

Model	AUC	Accuracy	Sensitivity	Specificity	F1
XGBoost	0.700 (0.637,0.764)	0.715 (0.668,0.758)	0.451 (0.348,0.559)	0.786 (0.737,0.829)	0.402
Random Forest	0.742 (0.685,0.799)	0.663 (0.615,0.709)	0.671 (0.563,0.763)	0.661 (0.606,0.712)	0.458
Logistic Regression	0.638 (0.569,0.707)	0.580 (0.531,0.629)	0.585 (0.477,0.686)	0.579 (0.523,0.633)	0.372
SVM	0.662 (0.592,0.733)	0.803 (0.761,0.840)	0.232 (0.154,0.334)	0.957 (0.928,0.975)	0.333
LightGBM	0.665 (0.597,0.733)	0.637 (0.588,0.684)	0.573 (0.465,0.675)	0.655 (0.600,0.706)	0.402
Ensemble	0.722 (0.660,0.783)	0.679 (0.631,0.723)	0.610 (0.502,0.708)	0.697 (0.644,0.746)	0.426

Based on the output of the machine learning pipeline, the predictive performance of the seven models was evaluated on the test set (N = 385). The Random Forest model achieved the best overall discrimination, with an AUC of 0.742 (95% CI: 0.685–0.799), followed by the Ensemble model (AUC = 0.722, 95% CI: 0.660–0.783) and XGBoost (AUC = 0.700, 95% CI: 0.637–0.764). Logistic Regression (AUC = 0.638, 95% CI: 0.569–0.707) and SVM (AUC = 0.662, 95% CI: 0.592–0.733) exhibited relatively lower discriminative ability. In terms of sensitivity, Random Forest correctly identified 67.1% of patients who experienced an in-hospital adverse event, while its specificity was 66.1%. Notably, SVM achieved the highest accuracy (80.3%) and specificity (95.7%) but very low sensitivity (23.2%), indicating a strong bias toward predicting the majority class.

Feature importance analyses revealed consistent patterns across models. In the Random Forest model, the top five predictors by mean decrease in accuracy were neutrophil percentage (18.08), albumin (11.17), bleeding volume (10.85), albumin/globulin ratio (10.74), and neutrophil absolute count (10.48). The SHAP analysis for the Random Forest model further confirmed these findings, with neutrophil percentage and albumin showing the highest mean absolute SHAP values. The consistency of these important features across multiple modeling approaches supports their potential relevance as clinical predictors.

Notably, the nine variables selected by LASSO were exactly those used in the modeling, and they consistently appeared among the top contributors in all feature importance assessments. This convergence suggests that the selected set captures clinically meaningful dimensions, including inflammatory status (neutrophil-related indices), nutritional and anemia-related parameters (albumin, red blood cell count, albumin/globulin ratio), uric acid, and surgical complexity (operation duration and bleeding volume). The Random Forest feature importance analysis identified neutrophil percentage (importance score: 18.08), albumin (11.17), bleeding volume (10.85), albumin/globulin ratio (10.74), and neutrophil absolute count (10.48) as the top five predictors. Collectively, these findings support the use of the Random Forest model as a potentially useful supportive risk-stratification tool that requires external and prospective validation before clinical implementation for predicting in-hospital adverse events, with a parsimonious set of predictors that are both interpretable and clinically accessible.

**Fig. 1:**
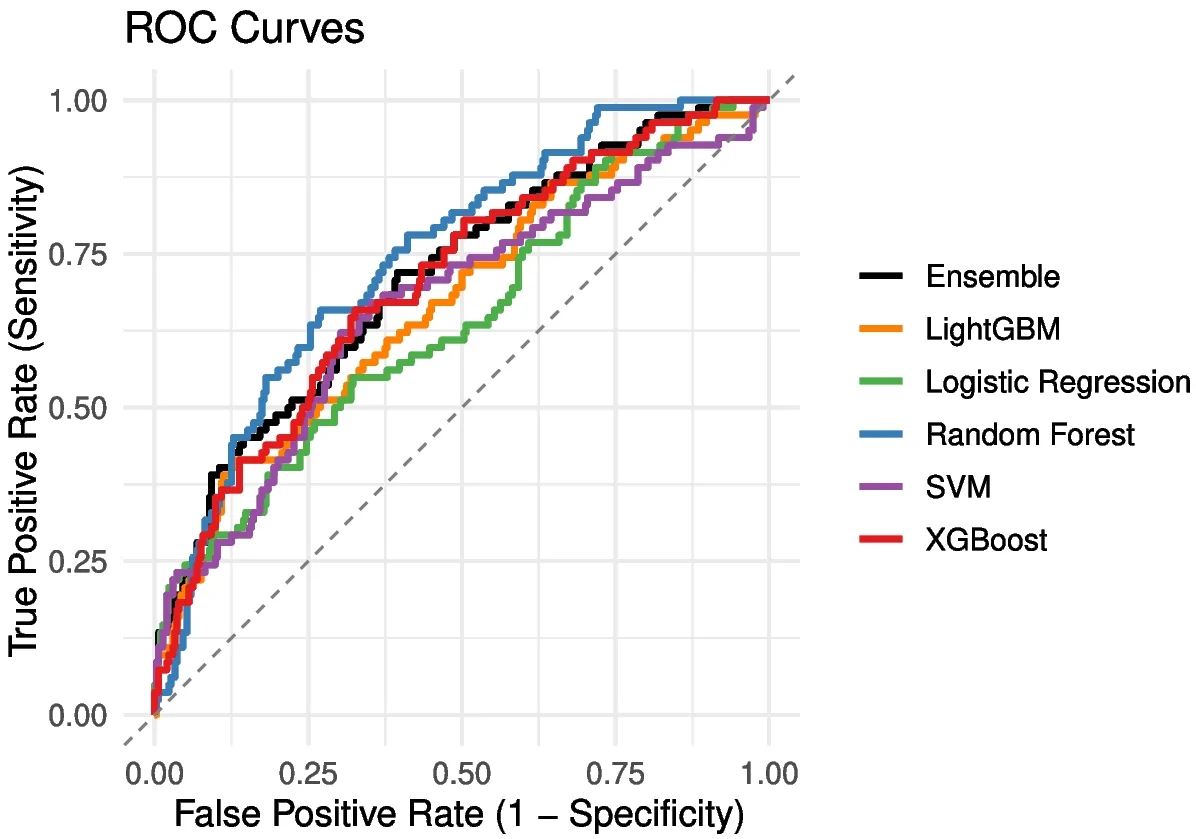
ROC curves for seven models. The Random Forest model achieved the highest AUC

**Fig. 2:**
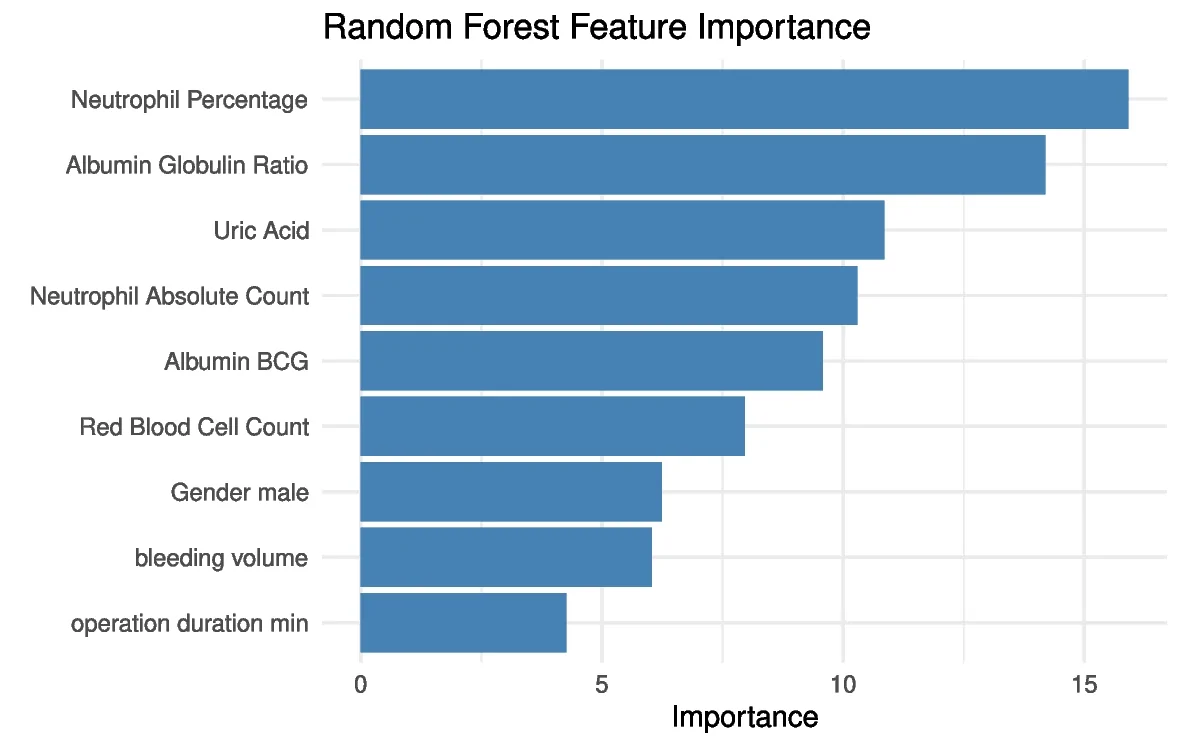
Importance summary plot for the Random Forest model

Figure 1 shows the ROC curves of all models. The Random Forest curve demonstrates the best discrimination ability, followed by the Ensemble model. Logistic Regression and SVM performed relatively poorly (Fig. 2).

### Decision curve analysis

**Fig. 3:**
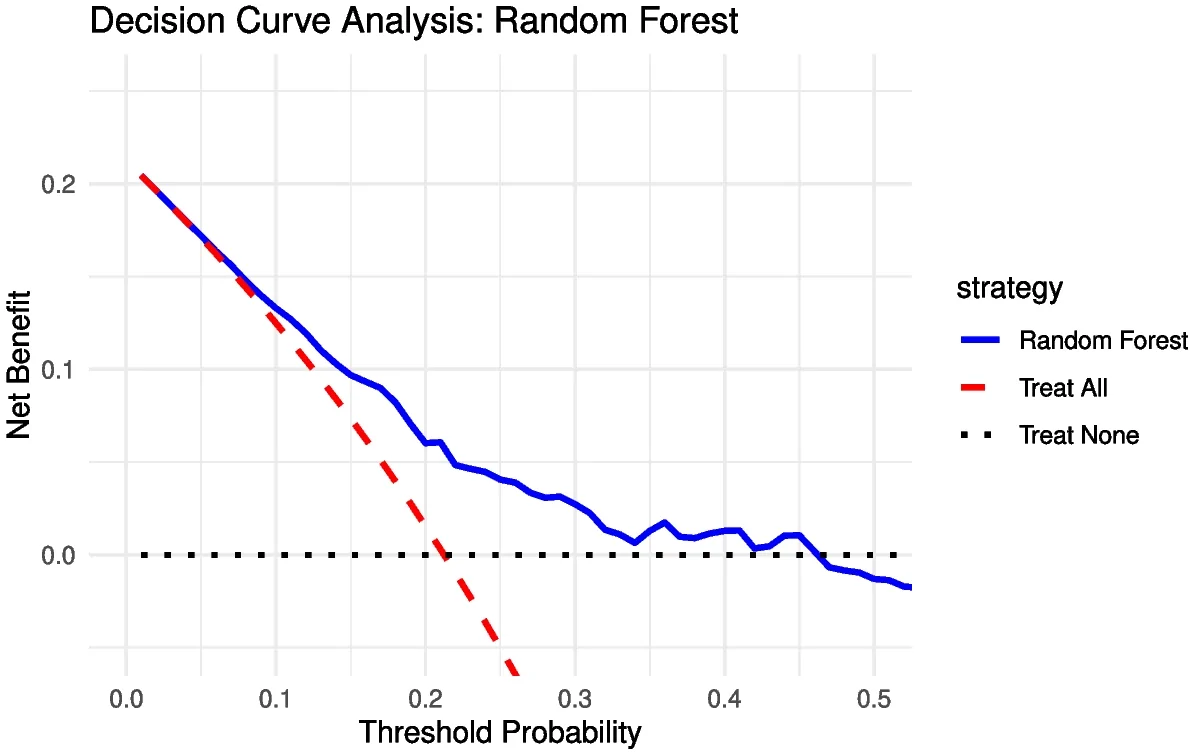
Decision curve analysis for the Random Forest model. The solid blue line represents the net benefit of the model across threshold probabilities. The dashed horizontal line represents the “treat none” strategy (net benefit = 0), and the solid gray line represents the “treat all” strategy

To evaluate the clinical utility of the Random Forest model, decision curve analysis (DCA) was performed across a range of clinically relevant threshold probabilities. As shown in Fig. 3, the model’s decision curve exceeded both the “treat all” and “treat none” strategies across threshold probabilities from approximately 0.05 to 0.40. The net benefit of the model exceeded 0.10 at threshold probabilities between approximately 0.10 and 0.25, indicating that the model provides clinically meaningful risk stratification that can guide intervention decisions without incurring unnecessary harm. These findings support the potential clinical utility of the model for identifying high-risk CHD patients who may benefit from more intensive monitoring or early intervention.

### Feature importance analysis

Causal forest method was used to analyze the causal effects of key predictor variables (Table 4).

**Table 4:** Causal Forest Analysis Results

Feature	ATE(%)	95%CI(%)	*p*-value	Conclusion
Red blood cell count	-6.76	[-12.14, -1.38]	0.014	Protective factor
Albumin/Globulin ratio	-4.79	[-9.98, 0.41]	0.071	Not significant
Albumin (BCG)	-4.04	[-9.37, 1.30]	0.138	Not significant
Bleeding volume	4.90	[-1.53, 11.33]	0.135	Not significant
Uric acid	-3.51	[-8.75, 1.74]	0.190	Not significant
Neutrophil percentage	3.77	[-1.95, 9.48]	0.196	Not significant
Neutrophil absolute count	2.32	[-3.42, 8.05]	0.429	Not significant
Operation duration	4.94	[-0.48, 10.35]	0.074	Not significant

Eight continuous LASSO-selected predictors were included in the causal forest analysis. Sex was not included as an exposure because it is a non-modifiable characteristic and was retained only as a predictive covariate. The analysis identified red blood cell count as a statistically significant protective factor, with an Average Treatment Effect of -6.76% (95% CI: -12.14 to -1.38, p = 0.014), indicating that higher red blood cell count is associated with reduced risk of in-hospital adverse events. The albumin/globulin ratio showed a borderline protective trend (ATE = -4.79%, 95% CI: -9.98 to 0.41, p = 0.071). Operation duration showed a borderline risk-increasing trend (ATE = 4.94%, 95% CI: -0.48 to 10.35, p = 0.074). Neutrophil percentage (p = 0.196), neutrophil absolute count (p = 0.429), albumin (BCG) (p = 0.138), uric acid (p = 0.190), and bleeding volume (p = 0.135) did not reach statistical significance.

### Sensitivity analyses

The primary analysis included 1,288 patients. Starting from 2,885 eligible patients, predictors with more than 60% missing observations were first removed, leaving 110 candidate predictors. Patients with more than 10% missing values across these predictors were then excluded. This step removed 1,204 patients and left 1,681 patients. A further 393 patients with at least one clinically implausible measurement were excluded from the primary analysis, resulting in the final sample of 1,288 patients.

To assess the influence of these exclusions, we constructed three nested sensitivity cohorts: (1) the complete eligible cohort without patient-level exclusion according to predictor missingness (*n=2,885*; 612 events); (2) patients with no more than 20% missing candidate predictors (*n=2,438*; 524 events); and (3) patients with no more than 10% missing candidate predictors (*n=1,681*; 350 events).

In these sensitivity analyses, the patient-level missingness criterion was applied before clinically implausible measurements were handled. Unlike the primary analysis, patients were not excluded because of an implausible measurement. Instead, only the affected measurement was recoded as missing, and the patient remained in the analysis. These recoded values, together with the originally missing values, were subsequently imputed using multiple imputation by chained equations. Consequently, the sensitivity analysis using the 10% missingness threshold included 1,681 patients, including the 393 patients who would have been excluded from the primary analysis because of clinically implausible measurements.

For each sensitivity cohort, the data were divided into training and held-out test sets using stratified random sampling at a ratio of 7:3. Missingness indicators were generated before imputation. Five multiply imputed datasets were created using predictive mean matching with five iterations; the imputation models were estimated using the training data, without using the held-out outcomes. Predictor selection was repeated within the training set using LASSO with $\lambda _{\min }$. Predictors selected in at least three of the five imputed datasets were retained for model development.

Seven modelling strategies were evaluated: XGBoost, random forest, logistic regression, support vector machine, LightGBM, and an ensemble model. Model tuning and selection of the classification threshold according to the Youden index were performed using training-set cross-validation. The held-out test set was used only for final performance evaluation. Predicted probabilities were pooled across the five imputed datasets.

As shown in Table 5, random forest and the ensemble model generally provided the highest discrimination. In the complete eligible cohort, random forest achieved an AUC of 0.6943 and the ensemble model achieved an AUC of 0.6922. When patients with more than 20% missing predictors were excluded, the corresponding AUCs increased to 0.7181 and 0.7145. Under the 10% missingness threshold, the ensemble model achieved an AUC of 0.7063, while random forest achieved an AUC of 0.6937. These findings indicate that the discriminatory performance of the models was generally maintained after retaining patients whose implausible measurements had previously resulted in complete-record exclusion, although performance varied according to the permitted amount of missing information.

**Table 5:** Model performance in the main sensitivity analyses (95% CI in parentheses)

Model	AUC	Accuracy	Sensitivity	Specificity	F1 score
*Complete eligible cohort: no patient-level missingness exclusion (**n=2,885**)*					
XGBoost	0.6389 (0.5926,0.6852)	0.5445 (0.5106,0.5781)	0.6486 (0.5752,0.7173)	0.5162 (0.4779,0.5543)	0.3785
Random forest	0.6943 (0.6518,0.7368)	0.5688 (0.5350,0.6021)	0.7243 (0.6540,0.7873)	0.5265 (0.4882,0.5645)	0.4181
Logistic regression	0.6288 (0.5824,0.6752)	0.5434 (0.5095,0.5769)	0.6486 (0.5752,0.7173)	0.5147 (0.4764,0.5529)	0.3780
Support vector machine	0.6419 (0.5965,0.6873)	0.6520 (0.6192,0.6838)	0.4757 (0.4019,0.5502)	0.7000 (0.6640,0.7342)	0.3690
LightGBM	0.6558 (0.6102,0.7013)	0.6254 (0.5922,0.6578)	0.5730 (0.4983,0.6453)	0.6397 (0.6023,0.6759)	0.3955
Ensemble	0.6922 (0.6499,0.7344)	0.5827 (0.5490,0.6158)	0.6919 (0.6200,0.7576)	0.5529 (0.5147,0.5907)	0.4149
*Patient-level missingness* $\le 20\%$ *(**n=2,438**)*					
XGBoost	0.6679 (0.6212,0.7146)	0.5841 (0.5474,0.6201)	0.6795 (0.6001,0.7519)	0.5583 (0.5166,0.5993)	0.4109
Random forest	0.7181 (0.6745,0.7616)	0.6047 (0.5682,0.6403)	0.7244 (0.6472,0.7928)	0.5722 (0.5306,0.6130)	0.4388
Logistic regression	0.6521 (0.6023,0.7019)	0.6936 (0.6587,0.7268)	0.4167 (0.3384,0.4982)	0.7687 (0.7320,0.8026)	0.3672
Support vector machine	0.6340 (0.5840,0.6840)	0.6443 (0.6084,0.6791)	0.5577 (0.4761,0.6371)	0.6678 (0.6277,0.7062)	0.4009
LightGBM	0.6827 (0.6379,0.7276)	0.4747 (0.4380,0.5116)	0.8654 (0.8016,0.9147)	0.3687 (0.3292,0.4096)	0.4128
Ensemble	0.7145 (0.6697,0.7592)	0.6867 (0.6517,0.7202)	0.5897 (0.5083,0.6678)	0.7130 (0.6742,0.7497)	0.4455
*Patient-level missingness* $\le 10\%$ *(**n=1,681**)*					
XGBoost	0.6858 (0.6288,0.7428)	0.7202 (0.6788,0.7590)	0.5000 (0.4013,0.5987)	0.7789 (0.7349,0.8187)	0.4291
Random forest	0.6937 (0.6388,0.7486)	0.6488 (0.6054,0.6905)	0.6509 (0.5522,0.7410)	0.6482 (0.5991,0.6952)	0.4381
Logistic regression	0.6761 (0.6183,0.7338)	0.5774 (0.5329,0.6209)	0.6981 (0.6013,0.7835)	0.5452 (0.4949,0.5949)	0.4100
Support vector machine	0.6183 (0.5594,0.6771)	0.6528 (0.6094,0.6943)	0.4151 (0.3202,0.5149)	0.7161 (0.6690,0.7599)	0.3346
LightGBM	0.6813 (0.6258,0.7369)	0.4881 (0.4436,0.5327)	0.8491 (0.7665,0.9112)	0.3920 (0.3437,0.4418)	0.4110
Ensemble	0.7063 (0.6532,0.7594)	0.6429 (0.5993,0.6847)	0.6415 (0.5426,0.7323)	0.6432 (0.5940,0.6903)	0.4304

## Web application for clinical decision support

To translate the predictive model into a practical clinical tool, we developed a web application using the R Shiny framework. The application provides an interactive interface for real-time assessment of in-hospital adverse event risk, allowing clinicians to input patient-specific clinical indicators and obtain immediate risk predictions.

### Application architecture and implementation

**Fig. 4:**
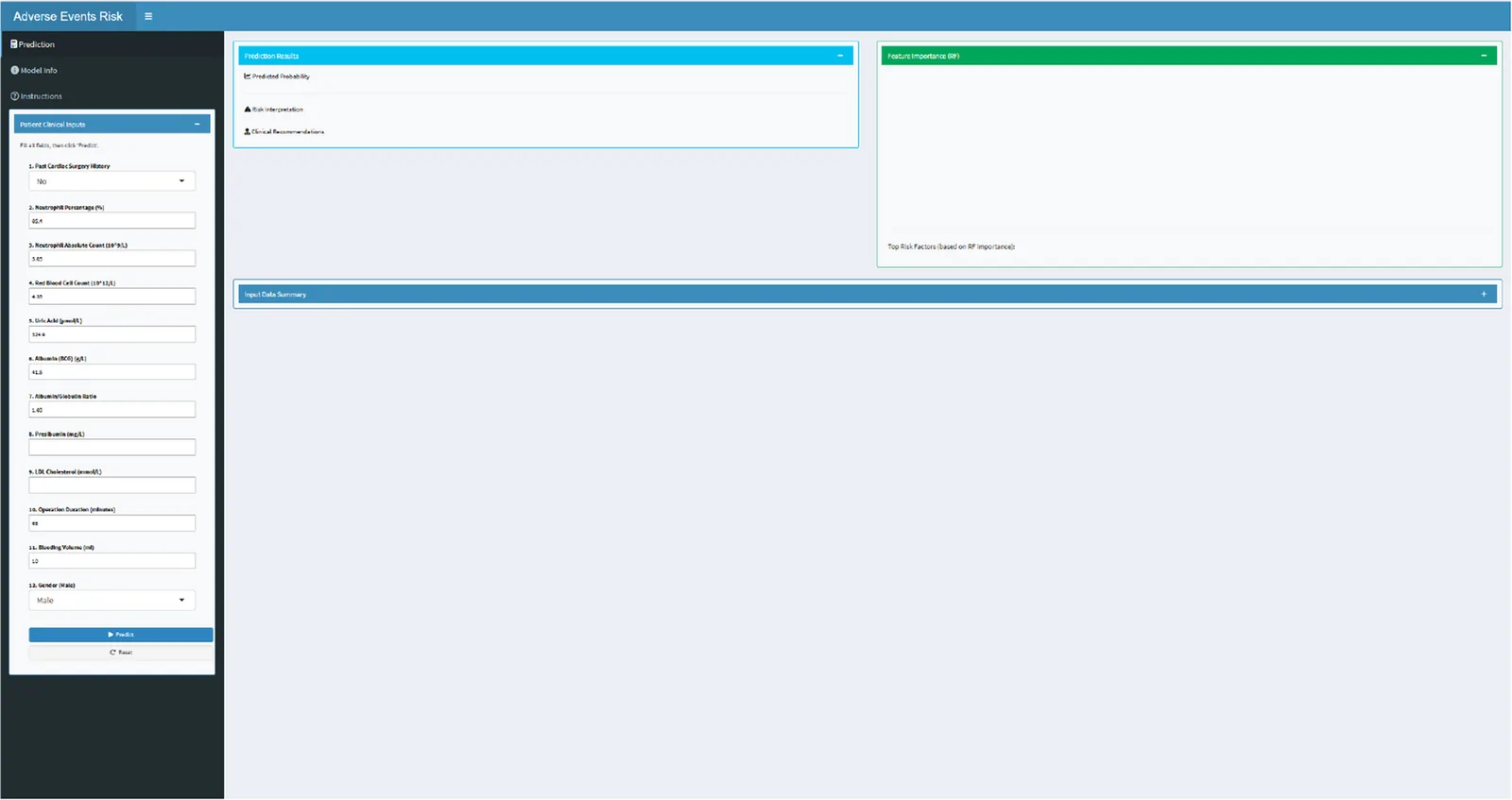
Main prediction interface of the web application. The left panel provides input fields for nine clinical indicators with default values set to training data medians. The right panel displays prediction results including risk probability, classification, and feature importance analysis

The application implements a modular three-panel design: (1) a prediction interface for data input and risk calculation, (2) a model information panel displaying technical specifications, and (3) an instructions panel providing usage guidelines. The interface was optimized for clinical workflow efficiency through compact layout design and rapid response times (Fig. 4).

The computational backend integrates the trained Random Forest model (AUC = 0.742). Upon user input submission, the application executes a streamlined prediction pipeline: data formatting, model inference, probability estimation, and risk stratification. Results display within two seconds, with model-predicted probabilities classified into three descriptive risk categories.

### Clinical functionality and output

**Fig. 5:**
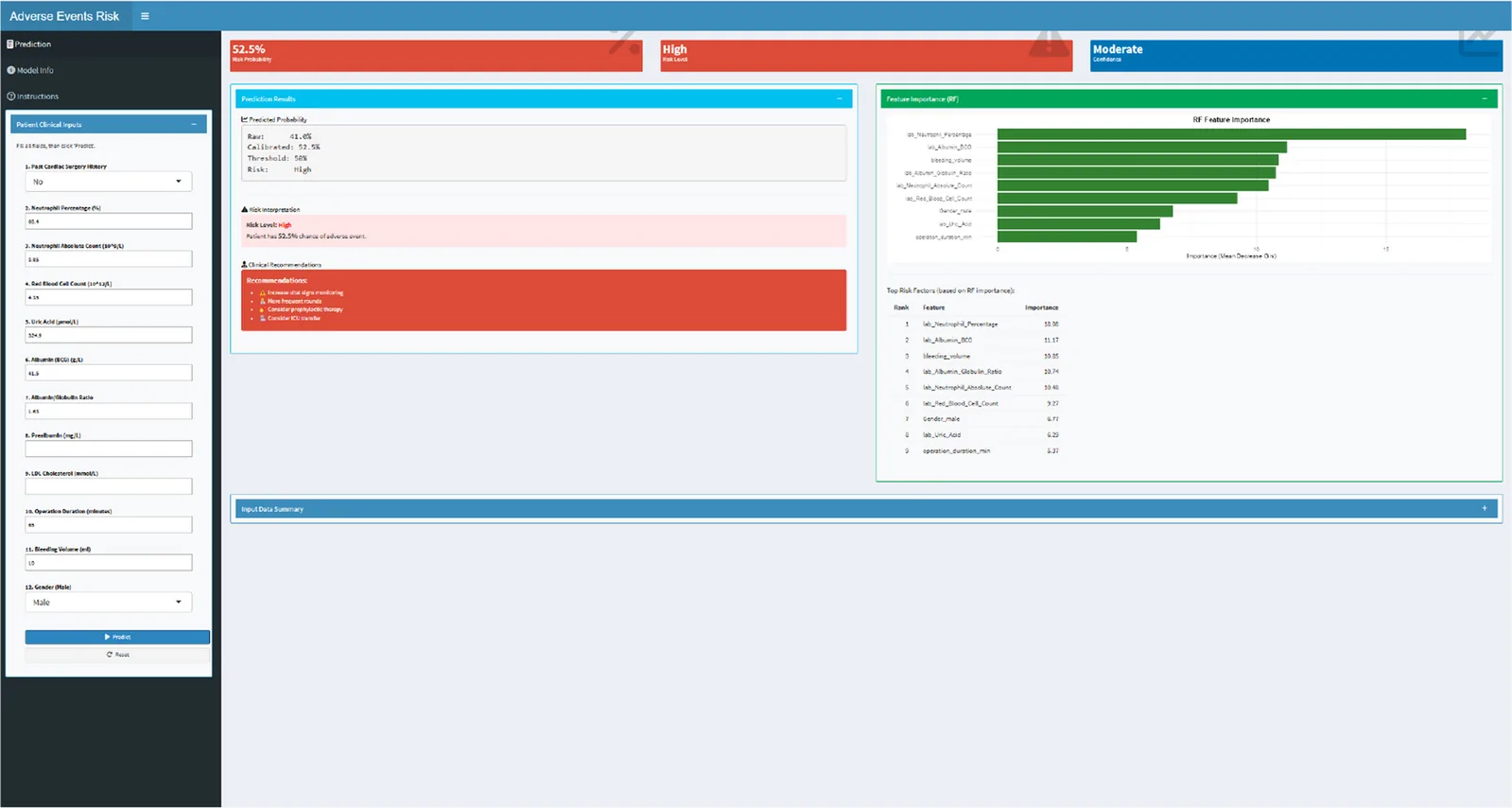
Prediction results panel showing risk assessment outputs. The dashboard displays key metrics, feature importance ranking, and clinical management recommendations tailored to the predicted risk level

The application generates comprehensive risk assessments combining quantitative predictions with clinical context. As shown in Fig. 5, the output includes calibrated probability estimates, three-tier risk classification, SHAP-based feature importance visualization, and risk-level-specific management recommendations. The feature importance analysis identifies neutrophil percentage (importance score: 18.08), albumin (11.17), and bleeding volume (10.85) as the most influential predictors in the model.

**Fig. 6:**
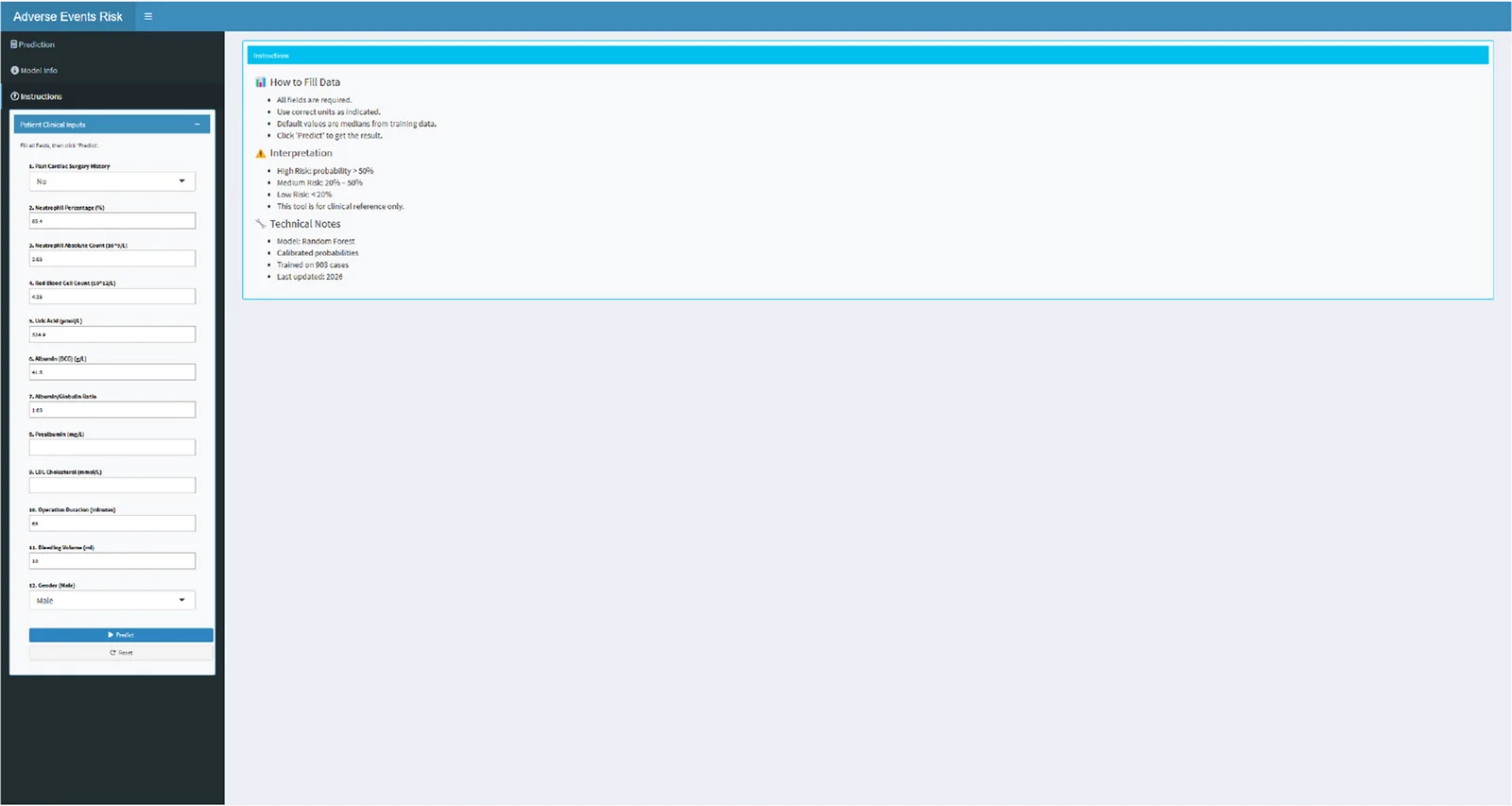
Model information panel providing technical details and performance metrics

Model transparency is provided through a dedicated information panel (Fig. 6) that documents technical specifications, performance metrics, and feature descriptions. This design addresses key requirements for clinical adoption of machine learning tools by maintaining interpretability and evidentiary foundation.

### Clinical utility and deployment

The application serves as a practical implementation of the predictive model, providing rapid, standardized risk assessment independent of individual clinician experience. It reduces cognitive burden through automated calculation while maintaining model interpretability through feature importance visualization. The tool is designed as a decision aid rather than a replacement for clinical judgment, with clear disclaimers regarding its supportive role in care pathways.

The system supports multiple deployment configurations including local execution within RStudio, institutional hosting on Shiny Server, and cloud-based deployment via shinyapps.io. The complete source code is provided as supplementary material, enabling adaptation and validation by other research institutions.

## Discussion

### Main findings

This study successfully developed a prediction model for in-hospital adverse events in CHD patients. The main findings are: (1) The Random Forest model achieved the best predictive performance with an AUC of approximately 0.74; (2) Neutrophil percentage, albumin, bleeding volume, albumin/globulin ratio, and neutrophil absolute count were the most important predictors; (3)Causal forest analysis suggested that higher red blood cell count was associated with a lower estimated risk of in-hospital adverse events ; (4) A web-based clinical decision support tool was developed to translate the model into practical clinical application.

### Model performance evaluation

This study compared 7 machine learning algorithms, with Random Forest achieving the best predictive performance (AUC=0.742). Random Forest, as an ensemble learning method based on decision trees, offers advantages including robust handling of non-linear relationships, built-in feature importance ranking, resistance to overfitting through ensemble averaging, and the ability to capture complex interactions among predictors.

Notably, although Random Forest had the highest AUC, its specificity (66.1%) was lower than some other models (e.g., Logistic Regression 95.7%), reflecting the sensitivity–specificity trade-off at the Youden-index-optimized threshold. This suggests that the classification threshold should be adjusted based on clinical priorities. The web application addresses this by providing raw probability estimates, allowing clinicians to select thresholds according to individual patient scenarios. We acknowledge that the AUC of approximately 0.74 represents moderate discriminatory performance. Several factors likely contribute: (1) the composite nature of the outcome – in-hospital adverse events encompass diverse conditions (cardiovascular complications, infections, bleeding, AKI, respiratory failure), each with potentially different risk factor profiles; (2) the absence of potentially important predictors such as echocardiographic parameters and medication data; (3) this AUC range (0.70–0.80) is consistent with other studies predicting composite in-hospital adverse events in cardiac populations. Thus, the model provides useful, though not definitive, risk discrimination.

This finding aligns with emerging trends in medical prognostics, where machine learning algorithms often outperform traditional statistical methods, particularly in handling complex, high-dimensional data and identifying non-linear relationships. Previous studies have shown that tree-based ensemble and gradient-boosting methods can provide useful discrimination in cardiovascular risk prediction [39–41]. Inflammatory and nutritional biomarkers, including neutrophil-related indices and albumin, have also demonstrated prognostic value in coronary and cardiac-surgery populations [42–45]. More recent studies have emphasized interpretable machine-learning frameworks and the translation of prediction models into clinically usable tools [46–49].

### Clinical translation and web application development

The successful development of the web-based decision support tool represents a critical advancement in translating predictive modeling research into clinical practice. The application provides a user-friendly interface that enables clinicians to input patient-specific data and obtain immediate risk assessments, addressing the gap between theoretical model performance and practical utility.

The application’s design incorporates several features to enhance clinical adoption. First, the compact interface layout optimizes screen real estate while maintaining data visibility, making it suitable for busy clinical environments. Second, the feature importance visualization based on SHAP values provides model interpretability, showing clinicians which factors contribute most to each individual prediction. This addresses the “black box” criticism often directed at machine learning models and enhances trust in the system’s outputs.

Third, the application provides context-specific clinical recommendations based on predicted risk levels. For high-risk patients (probability > 50%), it suggests intensified monitoring and potential preventive interventions; for medium-risk patients (20-50%), it recommends enhanced surveillance; and for low-risk patients (< 20%), it supports standard management protocols. These recommendations bridge the gap between statistical prediction and actionable clinical guidance.

Our analysis showed that prolonged operation duration had a borderline effect on adverse event risk (ATE = 4.94%, 95% CI: -0.48 to 10.35, p = 0.074). Possible mechanisms include prolonged anesthesia increasing cardiorespiratory complication risk and increased surgical trauma stress response. Similar associations between prolonged surgery and adverse outcomes are observed in other cardiac surgical contexts, highlighting the physiological burden of extended procedures [50]. Clinical implication is that for patients with expected long operation duration, thorough assessment of surgical necessity and patient tolerance should be performed, and staged surgery or minimally invasive approaches may be considered when appropriate. The web application quantifies this risk contribution, supporting surgical planning decisions.

#### Inflammatory markers

Neutrophil percentage and absolute count are commonly used inflammatory markers. Our study found significantly elevated neutrophil levels in the adverse event group, indicating a close relationship between inflammatory status and adverse events. Systemic inflammatory response can lead to endothelial dysfunction and coagulation abnormalities, increasing cardiovascular event risk [45].

The neutrophil-to-lymphocyte ratio (NLR) has emerged as a notable inflammatory marker in recent years. In this study, NLR was higher in the adverse-event group than in the non-adverse-event group (median 3.0 vs. 2.6, *p<0.001*)., consistent with previous research demonstrating its predictive and prognostic value in CHD and acute coronary syndrome (ACS) patients [44, 45, 51]. The NLR, calculated from routine blood tests, reflects the balance between innate and adaptive immunity, and its elevation indicates systemic inflammation [44]. The web application includes neutrophil parameters as key inputs, highlighting their importance in risk assessment.

#### Nutritional indicators

Albumin and prealbumin are important indicators for assessing patient nutritional status. Our study found significantly lower albumin levels in the adverse event group. Malnutrition is associated with decreased immune function, poor wound healing, and increased infection risk, which may be indirect predictors of adverse outcomes. This highlights the complex interplay between inflammation, nutrition, and cardiovascular health, where compromised nutritional status can exacerbate inflammatory processes and worsen patient prognosis. The application’s feature importance analysis quantifies the contribution of nutritional status to overall risk.

### Strengths and limitations

Several strengths and limitations of this study should be acknowledged. Strengths include a relatively large sample size (1,288 patients), comparison of multiple machine learning algorithms, combination of causal inference methods to explore risk factor effects, systematic data preprocessing including proper training/test set separation, and the development of a practical web application for clinical decision support.

The development of the web application represents a particular strength, as it demonstrates the practical translation of research findings into clinical tools. The application’s modular design allows for future enhancements, and its implementation of model interpretability features addresses a key barrier to clinical adoption of machine learning systems. The tool’s ability to provide immediate, individualized risk assessments with explanatory visualizations represents an advance over traditional risk scores.

The limitations include: single-center study with potential selection bias; retrospective study design; high missing rates for some laboratory indicators; lack of external validation; and causal forest analysis that only included a subset of key variables, potentially missing other important confounding factors.

Regarding the web application, current limitations include the need for manual data entry rather than electronic health record integration, lack of mobile device optimization, and absence of real-time model updating capabilities. Additionally, the application requires validation in prospective clinical settings to assess its impact on clinical outcomes and workflow integration.

Future research directions should include conducting multi-center, prospective studies to validate model performance, including more clinical variables such as medication information and imaging data, developing online prediction tools for clinical use, exploring deep learning methods to further improve prediction performance, and conducting subgroup analysis to identify high-risk population characteristics. Specifically for the web application, future work should focus on EHR integration, mobile-responsive design, multi-language support, and implementation of continuous learning mechanisms to incorporate new data over time.

## Conclusion

This study developed a prediction model for in-hospital adverse events in CHD patients. The Random Forest model demonstrates moderate predictive performance with an AUC of 0.742 and can serve as a risk assessment tool. Causal analysis identified red blood cell count as a protective factor (ATE = -6.76%, p = 0.014). Inflammatory indicators such as neutrophil percentage and count, and nutritional indicators including albumin and albumin/globulin ratio, are important predictors closely related to adverse event occurrence. The machine learning-based prediction model combined with causal inference analysis can provide scientific basis for early identification and intervention of in-hospital adverse events in CHD patients.

To translate these research findings into clinical practice, we developed a web-based decision support application that provides real-time risk assessment with model interpretability features. The application enables clinicians to input patient-specific data and obtain immediate, calibrated risk estimates with visual explanations of contributing factors. This tool bridges the gap between predictive modeling research and bedside decision-making, offering a practical implementation of evidence-based risk assessment. The application’s modular design supports future enhancements including EHR integration, mobile optimization, and continuous learning capabilities. By combining robust statistical methods with user-centered design, this research provides both theoretical insights and practical tools for improving patient outcomes in CHD management.

## Data Availability

The datasets generated and/or analyzed during the current study are available from the corresponding author on reasonable request.
